# Software Sensor for Activity-Time Monitoring and Fault Detection in Production Lines

**DOI:** 10.3390/s18072346

**Published:** 2018-07-19

**Authors:** Tamas Ruppert, Janos Abonyi

**Affiliations:** MTA-PE “Lendület” Complex Systems Monitoring Research Group, Department of Process Engineering, University of Pannonia, Egyetem u. 10, POB 158, H-8200 Veszprém, Hungary; ruppertt@fmt.uni-pannon.hu

**Keywords:** recursive estimation, performance monitoring, indoor positioning system, paced conveyor, early warning systems

## Abstract

Industry 4.0-based human-in-the-loop cyber-physical production systems are transforming the industrial workforce to accommodate the ever-increasing variability of production. Real-time operator support and performance monitoring require accurate information on the activities of operators. The problem with tracing hundreds of activity times is critical due to the enormous variability and complexity of products. To handle this problem a software-sensor-based activity-time and performance measurement system is proposed. To ensure a real-time connection between operator performance and varying product complexity, fixture sensors and an indoor positioning system (IPS) were designed and this multi sensor data merged with product-relevant information. The proposed model-based performance monitoring system tracks the recursively estimated parameters of the activity-time estimation model. As the estimation problem can be ill-conditioned and poor raw sensor data can result in unrealistic parameter estimates, constraints were introduced into the parameter-estimation algorithm to increase the robustness of the software sensor. The applicability of the proposed methodology is demonstrated on a well-documented benchmark problem of a wire harness manufacturing process. The fully reproducible and realistic simulation study confirms that the indoor positioning system-based integration of primary sensor signals and product-relevant information can be efficiently utilized in terms of the constrained recursive estimation of the operator activity.

## 1. Introduction

In the age of digital transformation, human operators are still applied in manufacturing processes. The Operator 4.0 concept aims to create human-cyber-physical production systems (H-CPPS) that improve the abilities of the operators’ thanks to the dynamic interaction between humans and production systems [1]. Smart sensors are key components of CPPS solutions [2]. Model-based production control and performance monitoring require accurate information concerning the activity times of the operators. Handling human factors is a challenging problem in terms of both cellular manufacturing [3] and human-robot interaction [4]. Usually operator activity is monitored by computer vision-based motion detection systems and Radio Frequency IDentification (RFID)-based object tracking [5]. Context-aware systems require unobtrusive sensors to track each step of the performed task and present the worker with the information needed at any given moment [6]. As wearable sensors are becoming more common, their utilization is also becoming more attractive [7]. However, hand motion-based activity recognition is still challenging [8] and requires the application of advanced machine learning algorithms [9]. As this brief overview shows as well, the tracking of operator activity is a difficult, highly infrastructure-demanding task which should utilize information stream fusion approaches to improve the robustness of the algorithms [10].

Tracing hundreds of primary activities is critical due to the enormous variability and complexity of products. As every operator performs sequentially a specific set of actions over a period of time, our goal is to develop a sensor system that continuously estimates the time consumption of these elementary activities. We model the time consumptions of these actions by activity time models and compare the estimated activity times to the performance of operators and generate early warnings when their productivity decreases.

For the cost-effective and robust measurement of assembly times, sensors were developed to record the timestamps related to the activity when the components are pushed into the fixtures by operators. As the activities of operators depend on the type and number of the built-in components, the production flow is tracked by an indoor positioning system (IPS). For the localization of the products and identification of the status of the conveyor system, Ultra-Wide band (UWB) IPS technology is applied with its low energy demand for transmitting information over a broad bandwidth (>500 MHz) and its accuracy with the range of 30–50 cm, which is significantly better than the one-meter uncertainty of Bluetooth Low Energy (BLE)-based solutions [11,12].

To integrate measurements originating from the IPS, a varying number (10–100) of active or passive fixture sensors, and other information sources of the production management system, a multi-sensor data fusion (MSDF) algorithm has been developed. Multiple sensors provide redundancy enabling the robust recursive estimation of the unmeasured primary activity times of the operators. To constrain the model parameters to lie within a reliable region and incorporate important *a priori* knowledge concerning the activity times, the estimated parameters were optimally projected on to a set of linear constraints by quadratic programming [13]. This central estimation enhances the confidence of the nominal model which improves the performance of fault detection based on the reconciliation of the local measurements.

The development of the proposed fault-detection algorithm is motivated by the analysis of an industrial wire harness manufacturing process which is a typical complex modular product manufacturing system [14,15]. To ensure our results are fully reproducible, only openly available information on wire harness manufacturing technologies was utilized during the development of the realistic case study. To stimulate further research, the resultant algorithm of the developed model of the manufacturing system and the details of the products and sensor placements are publicly available on the website of the authors (https://www.abonyilab.com/soft-sensors).

The remaining part of the paper is structured as follows. The developed IIoT-based sensor system is shown in Section 2. The applicability of the proposed activity-time estimation algorithm is demonstrated in Section 3. Based on the findings and discussions reported there, conclusions are drawn in Section 4.

## 2. Software Sensor for Activity-Time Monitoring

In the present section, first the conveyor and the modular production systems are characterized, then the fixture sensors and the indoor positioning system as information sources are described. This is followed by the mathematical formulation of the multi sensor data fusion-based recursive estimation model and finally by the local estimation and monitoring with regard to the activity times of operators.

### 2.1. Problem Definition—Evaluation of Activity Times on the Paced Conveyor

**Figure 1:**
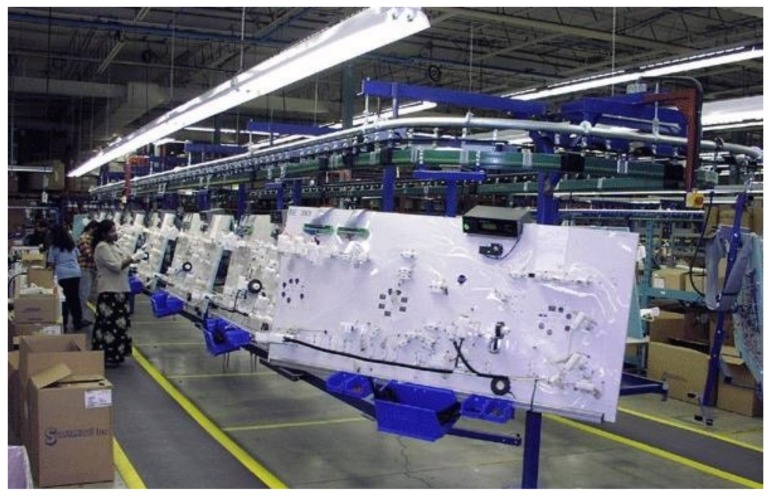
The wire harness paced assembly conveyor (often referred to as a rotary) contains assembly tables consisting of connector and clip fixtures [18].

The development of the proposed fault-detection algorithm is motivated by the analysis of an industrial wire harness manufacturing process. Wire harnesses are produced by a typical complex modular production system [14,15]. The crucial part of the studied wire harness manufacturing system is a similar conveyor system as shown in Figure 1. The motion of the conveyor is paced and cyclic in nature. At the beginning of the cycles, every station proceeds to the next position. The operators might work ahead of schedule or be delayed. According to the open-station concept, when the operator does not finish his or her job, he or she can move with the product to the next station to reduce the backlog. When the operator completes the task before the end of the cycle time, he or she can work ahead of schedule [16]. Production stops when the delay exceeds a critical limit. Contrary to this open station-type operating strategy, close-station production is referred to when the operator must stop the conveyor even in the event of a minor delay [17].

The key idea is that in the case of modular production, the expected activity times are estimated based on the Bill of Materials (BoM) of the manufactured products. The manufacturing is modular meaning that the products $p_{1},\ldots ,p_{N_{p}}$ are built from the set of modules $m_{1},\ldots ,m_{N_{m}}$ [19]. The structures of the products are defined by a P-matrix (also referred to as a binary/logical matrix) consisting of $N_{p}$ rows and $N_{m}$ columns, and the element $p_{i,j}$ of P is set to one when the $p_{i}$-th type of product contains the $m_{j}$-th module (otherwise it is 0). The calculation of the theoretical activity times is estimated based on which $a_{1},\ldots ,a_{N_{a}}$ activities are needed to be performed and which $c_{1},\ldots ,c_{N_{c}}$ components should be built in at the $w_{1},\ldots ,w_{N_{w}}$ workstations. This information is represented in the logical matrix M that contains the activities required to produce a given product. As is shown in Table 1, the C matrix stores which components are built in in each activity, while the W matrix assigns activities to the workstations. The specific activity times and factors influencing them were determined based on expert knowledge [15] as presented in Table 2. The matrix T provides information on the category of the activity describing how the activities are classified into the activity types $t_{1},\ldots ,t_{N_{t}}$. The sequence of the products is represented by a π vector of the labels of the types, so $\pi \left(k\right)=p_{j}$ states that type product $p_{j}$ started to be produced during the *k*-th production cycle.

To ensure fully reproducible results, only openly available information on wire harness manufacturing technologies was utilized during the development of this case study. To stimulate further research, the resultant algorithm of the developed model of the production system and the details of the products and sensor placements are publicly available on the website of the authors (https://www.abonyilab.com/soft-sensors).

Based on the data published in [14,15], the number of types of products $N_{p}$ is assumed to be 64 and defined as the combination of $N_{m}=7$ modules: base module $m_{1}$, left- or right-hand drive $m_{2}$, normal/hybrid $m_{3}$, halogen/LED lights $m_{4}$, petrol/diesel engine $m_{5}$, 4 doors/5 doors $m_{6}$, and manual or automatic gearbox $m_{7}$. The number of activities/tasks $N_{a}$ is defined as 654 and categorized into $N_{t}=16$ types of activities. The time consumptions of these activities are approximated using a direct proportionality approach with regard to the primary activities (see Table 2). During the activities involved in the production of the base harness 115 different part families (component types, $N_{c}$) are built in (among these $C_{t}=162$ terminals, $C_{b}=63$ bandages, $C_{c}=25$ clips, and $C_{w}=89$ wires). The conveyor consists of 10 workstations (tables, $N_{w}$). For every table (workstation) one operator is assigned, therefore, $N_{o}=10$.

Hereinafter, the term primary activity time denotes the estimated average period of time required for a certain type of activity to be performed, while the term local activity time refers to the time period required by a specific operator at the *w*-th workstation to perform the activity in question. The structure of the developed production-monitoring model is determined by the available information [15]. The proposed matrix-based mathematical formulation is beneficial as it allows the compact estimation of the individual ${\hat{y}}_{i}^{w}\left(k\right),i=1,\ldots ,N_{a}$ activity times in every *k* cycle step (discrete time):

(1) $${\hat{y}}_{i}^{w}\left(k\right)=\left[t_{i},c_{i}\right]x^{w}\left(k\right)\;,$$

as the time consumption of the *i*-th activity depends on how many elementary activities of a given type should be performed (represented as $t_{i}$ which is the *i*-th row of the matrix T), the number of built in components (the row vector $c_{i}$ is the *i*-th row of the matrix C) and the ’efficiency’ of the operator $x^{w}\left(k\right)$, which is the vector of the estimated local activity times. Therefore, the aim of our investigation is to provide a continuous local estimate of this state vector and its workstation independent x(k) version providing a reference value and the opportunity for the isolation of operator-independent problems.

### 2.2. Fixture Sensor- and Indoor Positioning System-Based Activity-Time Measurements

**Figure 2:**
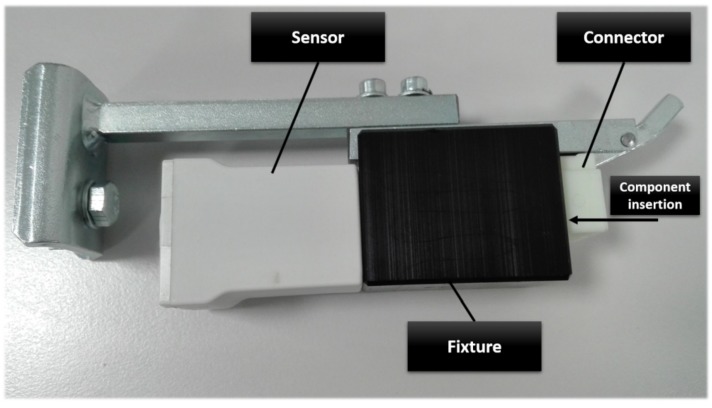
The designed connector fixture sends timestamps when the operator inserts a component into a fixture.

**Figure 3:**
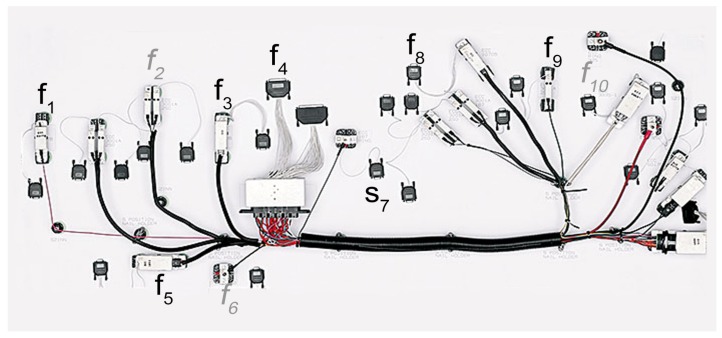
Illustration of the distribution of the fixtures on an assembly table. As the fixtures move according to the tables of the conveyor system, the fixtures are identically placed at every workstation. The fixtures labeled with gray text are inactive as there are no related activities at the depicted workstation.

To measure the activity times, fixture sensors were designed as depicted in Figure 2. The fixture-based activity sensors generate timestamps when the component is inserted into the fixture. The sensors on an illustrated assembly table are shown in Figure 3.

The fixtures were positioned based on how the measurable activities at the workstations are distributed. For example, the sensor $f_{1}$ sends a timestamps when the operator inserts the component $c_{1}$ which represents the starting time of the first activity $a_{1}$. Details concerning the placement of the sensors are given in Table 3.

The activity-dependent sequence of the timestamps recorded by the active sensors in the *k*-th cycle of the conveyor is represented by vector $s\left(k\right)={\left[s_{1}\left(k\right),\ldots ,s_{j}\left(k\right)\ldots ,s_{N_{s}}\left(k\right)\right]}^{T}$ which serves as the raw input of the performance-monitoring algorithm.

**Figure 4:**
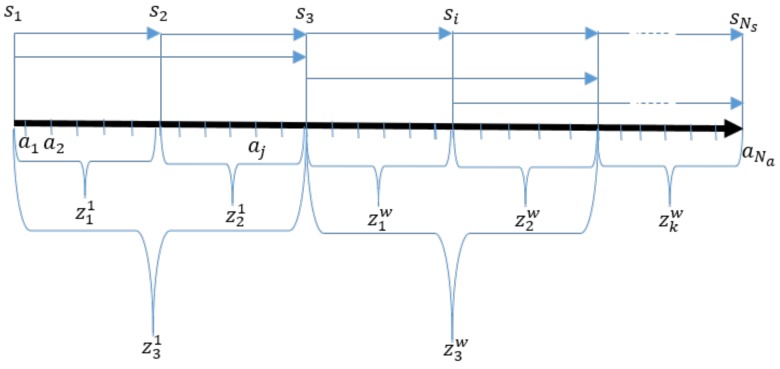
The concept of activity time measurements. The differences between the timestamps ($s_{i}$) define the time period required by a set of activities ($a_{j}$), the totals of which are considered as measured variables at each workstation ($z_{i}^{w}$), where *w* represents the index of the workstation.

As is shown in Figure 4, two timestamps clasp a set of activities, therefore, the $z_{i}^{w}\left(k\right)=s_{\beta (i)}^{w}\left(k\right)-s_{\alpha (i)}^{w}\left(k\right)$ difference between any two timestamps provides the sum of the activity times that are situated between the two sensors. If the timestamps $s_{\alpha (i)}^{w}\left(k\right)$ measures the start of the first activity at the *w*-th workstation, the station time of the *w*-th workstation can be measured as $z_{i}^{w}\left(k\right)=s_{\alpha (i)}^{w}\left(k+1\right)-s_{\alpha (i)}^{w}\left(k\right)$. Based on this concept, a set of measurements can be defined for the workstations $z^{w}\left(k\right)={\left[z_{1}^{w}\left(k\right),\ldots ,z_{i}^{w}\left(k\right),\ldots z_{l_{w}}^{w}\left(k\right)\right]}^{T}$ which is much more interpretable and applicable information with regard to activity-time monitoring than the s(k) values of the raw measurements.

To put $z^{w}\left(k\right)$ into context, the information on which products are assembled at each station and the details of the activities that are assigned to the measured time interval $z_{i}^{w}\left(k\right)$ are required.

The assignment of the activities and the measured time intervals are represented by a set of logical matrices $S^{w}$ (see Table 1). In the case of modular production the set of activities $q_{a}=Mp_{p}^{T}$ should be calculated based on which modules are included in the produced *p*-th product (represented as $p_{p}$ which is the *p*-th row of the product-module matrix P) and whose activities are required to produce the modules (such information is stored in the relation matrix M). The activities that are assigned to the $z^{w}\left(k\right)$-th intervals are defined by the operation $diag\left(q_{a}\right)S^{w}$.

$T^{T}diag\left(q_{a}\right)S^{w}$ groups the activities according to activity types, while the number of components installed over a specific time interval is calculated as $C^{T}diag\left(q_{a}\right)S^{w}$, which can also be grouped by activity types according to $\left(T^{T}C>0\right)C^{\prime }diag\left(q_{a}\right)S^{w}$. Based on the proposed matrix-type representation, the estimated time intervals at the *w*-th workstation can be calculated as:

(2) $${\hat{z}}^{w}\left(k\right)=\left[T^{T}diag\left(q_{a}\right)S^{w}\;,\left(T^{T}C>0\right)C^{T}diag\left(q_{a}\right)S^{w}\right]x^{w}\left(k\right)=H^{w}\left(k\right)x^{w}\left(k\right)$$

The model equation $z^{w}\left(k\right)=H^{w}\left(k\right)x^{w}\left(k\right)+e^{w}$ and the related measurements $z^{w}\left(k\right)$ can be used for the continuous estimation of the vector of operator efficiencies (namely estimated local activity times), $x^{w}\left(k\right)$, where $e^{w}\left(k\right)$ is assumed to be a serially uncorrelated white-noise vector of observational errors with covariance matrix $R^{w}\left(k\right)$.

As $H^{w}\left(k\right)$ depends on the actual product, which product is produced at the *w*-th workstation must be tracked. For the localization of the products and identification of the status of the conveyor system, an Ultra-Wide band (UWB) Indoor Positioning System (IPS) technology with its low energy demand for transmitting information over a broad bandwidth (>500 MHz) and accuracy within the range of 30–50 cm, which is significantly better than the uncertainty of one meter that the BLE based solutions posses [11,12], was applied.

In comparison with outdoor environments, sensing location information in indoor environments requires higher precision which is a more challenging task because various objects reflect and disperse signals. Ultra-Wideband (UWB) is an emerging technology in the field of indoor positioning [20] that has shown better performance compared to others [21] even in the presence of severe multipath [22,23]. Depending on the positioning technique, the angle of arrival (AOA), the signal strength (SS), or time delay information can be used for positioning [12]. Received signal strength (RSS) UWB positioning methods also can be divided into Time of Arrival (ToA), Angle of Arrival (AoA) and Received Signal Strength (RSS)-based systems [24].

**Figure 5:**
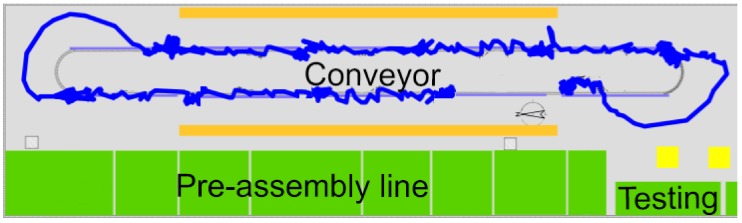
Illustration of how the internal positioning system (IPS) tracks a product in the conveyor at the table. The tracking is accurate and nicely depicts the rotations of the table at the edges of the conveyor.

The concept of identification of the products at workstations to extract product-relevant information from the Bill of Materials (BoM) and other structured information sources are widely used to support production management [25], value stream mapping [26], and Industrial Internet of Things (IIoT)-based lifecycle management [27]. In the developed system the IPS beacons are mounted to the flat wire-harness and the raw signals of the receivers (shown in Figure 5) are processed to assign the cables to the workstations.

### 2.3. Multi-Sensor Data Fusion-Based Recursive Estimation

**Figure 6:**
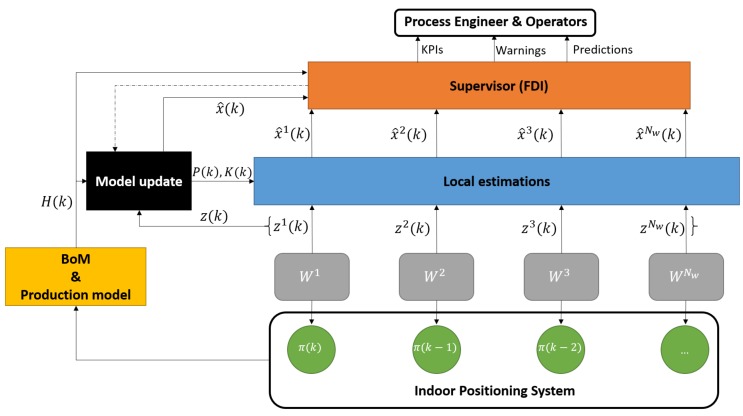
The sensor fusion-based architecture of the proposed monitoring system.

Multiple sensors provide redundancy which enables the robust recursive estimation of the unmeasured primary activity times of the operators. Therefore, the estimation problem is defined as a sensor-fusion task [28]. The presented sensor fusion algorithm combines all sensory and production data such that the estimates of the activity times have less uncertainty than would be possible when these sources were used individually. The elements of the monitoring system are structured as shown in Figure 6.

The fusion center receives and synchronizes all the $z^{w}\left(k\right),w=1,\ldots ,N_{w}$ measured time intervals and the related $H^{w}\left(k\right),w=1,\ldots ,N_{w}$ time-variable regressors, which means all data collected from the workstations are time-stamped and arranged according to *k*-th cycle of the conveyor:

(3) $$z\left(k\right)=\left[\begin{array}{c} z^{1}\left(k\right)\\ \vdots \\ z^{N_{w}}\left(k\right) \end{array}\right]\;,H\left(k\right)=\left[\begin{array}{c} H^{1}\left(k\right)\\ \vdots \\ H^{N_{w}}\left(k\right) \end{array}\right]\;,$$

The linear structure of the developed production-monitoring model (see Equation (1)) is adequate for the studied problem as the time consumption of the activities linearly depend on how many elementary activities should be performed and what is the number of the built in components [15]. When a linear sensor-fusion model is assumed, the previously presented linear time-variant model can be represented as

(4) z(k)=H(k)x(k)+e(k),

where the e(k) noise vector of the fused observations consists of the $e^{w}\left(k\right)$ serially uncorrelated white-noise vectors of observational errors at the workstations, $e\left(k\right)={\left[{\left(e^{1}\left(k\right)\right)}^{T},\ldots ,{\left(e^{N_{w}}\left(k\right)\right)}^{T}\right]}^{T}$.

When the observation errors of the workstations are assumed to be independent, the covariance of the e(k) noise vector is a block diagonal matrix defined as $R=diag\left(R^{1},\ldots ,R^{N_{w}}\right)$.

The central estimation enhances the confidence of the nominal model which improves the performance of fault detection based on the reconciliation of the local measurements [29].

Based on k=1,…,N synchronized z(k) and H(k) observations the objective function of the central estimation problem can be formalized as:

(5) $$\hat{x}\left(N\right)=\arg\min_{x}V_{N}\left(x\right)\;\;V_{N}\left(x\right)=\frac{1}{N}\sum_{k=1}^{N}{\left[z(k)-H(k)x\right]}^{T}Q\left[z(k)-H(k)x\right]$$

When the positive-definite weighting matrix Q is defined as $Q={\left(R\right)}^{-1}$, the estimation is equivalent to the maximum-likelihood cost function [30].

The covariance matrix of the estimation error $\tilde{x}\left(k\right)=\hat{x}\left(k\right)-x\left(k\right)$ is:

(6) $$E\left(\tilde{x}\left(N\right){\tilde{x}}^{T}\left(N\right)\right)=P^{*}\left(N\right)={\left[\sum_{k=1}^{N}H^{T}\left(k\right)R^{-1}H\left(k\right)\right]}^{-1}$$

The recursive estimation of the primary activity times x(k) is similar to the state estimation algorithm which assumes the following Gauss-Markov (GM) process:

(7) $$\begin{array}{ccc} x(k) & = & A^{*}\left(k\right)x\left(k-1\right)+\eta \left(k-1\right),\;\;\eta \left(k\right)=N\left(0,Q_{x}\right) \end{array}$$

(8) $$\begin{array}{ccc} z(k) & = & H(k)x(k)+e(k),\;\;e(k)=N(0,R) \end{array}$$

where η(k) noise vector and its $Q_{x}$ covariance matrix represents the uncertainty of the unknown and time-varying parameters and $A^{*}\left(k\right)$ stands for the state transition matrix of this random process.

The recursive estimation consists of prediction and correction steps as follows.

At the prediction step the state vector and its covariance matrix is calculated based on information available at the k-1 time instant:

(9) $$\begin{array}{ccc} \hat{x}\left(kk-1\right) & = & \hat{x}\left(k-1\right) \end{array}$$

(10) $$\begin{array}{ccc} P^{*}\left(kk-1\right) & = & P^{*}\left(k-1\right)+Q_{x} \end{array}$$

The correction step utilizes the measured z(k) measurements at the correction the estimated state variables by the $e\left(k\right)=\left[z\left(k\right)-H\left(k\right)\hat{x}\left(kk-1\right)\right]$ prediction error, with the K(k) time-varying Kalman gain updated based on the refreshed $P^{*}\left(k\right)$ covariance matrix:

(11) $$\begin{array}{ccc} \hat{x}\left(k\right) & = & \hat{x}\left(kk-1\right)+K\left(k\right)\left[z\left(k\right)-H\left(k\right)\hat{x}\left(kk-1\right)\right] \end{array}$$

(12) $$\begin{array}{ccc} K(k) & = & P^{*}\left(kk-1\right)H^{T}\left(k\right){\left[R+H\left(k\right)P^{*}\left(kk-1\right)H^{*T}\left(k\right)\right]}^{-1} \end{array}$$

(13) $$\begin{array}{ccc} P^{*}\left(k\right) & = & P^{*}\left(kk-1\right)-K\left(k\right)H\left(k\right)P^{*}\left(kk-1\right) \end{array}$$

### 2.4. Local Estimation and Monitoring of the Primary Activity Times

To constrain the model parameters to lie within a reliable region and incorporate important *a priori* knowledge of the activity times, the estimated parameters were optimally projected on to the set of linear constraints by quadratic programming [13].

The local (operator-related) projection of the unconstrained estimate $\hat{x}\left(k\right)$ can be considered as a quadratic programming problem:

(14) $$\hat{x^{w}}\left(k\right)=arg\min_{x(k)}{\left[x\left(k\right)-\hat{x}\left(k\right)\right]}^{T}Q_{p}\left[x\left(k\right)-\hat{x}\left(k\right)\right]$$

subject to:

(15) $$\begin{array}{ccc} A_{e}^{w}\left(k\right)x\left(k\right) & = & b_{e}^{w}\left(k\right) \end{array}$$

(16) $$\begin{array}{ccc} L^{w}x\left(k\right) & \leq & c^{w} \end{array}$$

(17) $$\hat{x}{\left(k\right)}^{c}=\hat{x}\left(k\right)-P^{*}\left(k\right)H_{j}^{T}\mu_{j}-P^{*}\left(k\right)L_{j}^{T}\lambda_{j}$$

where $\hat{x}\left(k\right)$ denotes the unconstrained solution, $\hat{x}{\left(k\right)}^{c}$ denote the constrained solution, $A_{e}^{w}\left(k\right)$ and $b_{e}^{w}\left(k\right)$ define the linear equality constraints, while $L^{w}\left(k\right)$ and $c^{w}\left(k\right)$ represent the linear inequalities. $\mu_{j}$ and $\lambda_{j}$ are vectors of Lagrange multipliers associated with equality and inequality constraints. This formulation ensures the optimal (least squares correction) when $Q_{p}={\left(P^{*}\left(k\right)\right)}^{-1}$. When $Q_{p}$ denotes the identity matrix an orthogonal projection is obtained. Assuming the constraints are true, parameter bias can never be increased [13].

The following section demonstrates how the estimated and expected primary activity times are used for production monitoring.

## 3. Wire Harness Case Study

### 3.1. Online Monitoring of Operator Performance

**Figure 7:**
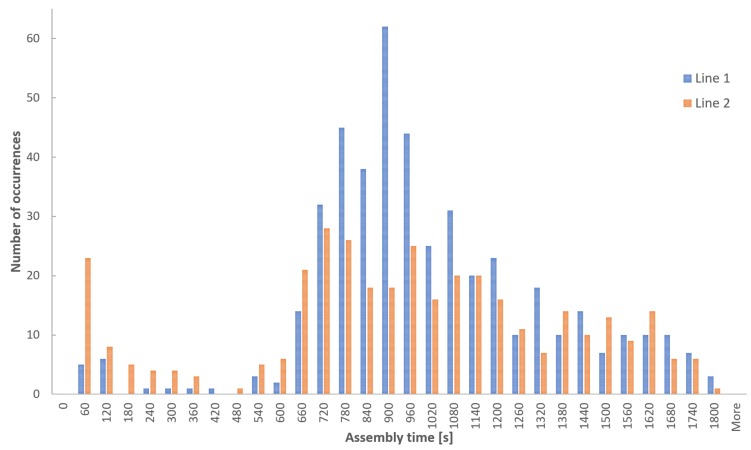
The histogram of measured processing times in two different conveyors (production lines). The histograms indicate that the distribution of the sensor-delivered processing times can be decomposed into normal distribution functions according to different products.

To validate the reliability of the proposed model, the distribution of the activity times collected from real production lines was studied. As is illustrated in Figure 7 the distribution of the assembly times can be broken down into several Gaussian-type distributions.

The identifiability of the model is determined by the rank of the covariance matrix $P^{*}\left(N\right)$. When the rank is smaller than the number of measurements (which occurs when the individual performance of operators is estimated at a specific workstation) only a subset of the parameters is identifiable.

The information content of the available data can be evaluated based on the eigenvalues or determinant of the covariance matrix $P^{*}\left(N\right)$. The tools of D-optimal experimental design that tries to maximize the determinant of $F^{*}\left(N\right)$ which is identical to the minimization of the determinant of $P^{*}\left(N\right)$ where utilized.

(18) $$F^{*}\left(N\right)={\left(P^{*}\left(N\right)\right)}^{-1}=\sum_{k=1}^{N}H^{T}\left(k\right)R^{-1}H\left(k\right)$$

When only one product is produced, H(k) does not change in terms of time. In this case, the set of the identifiable parameters for a given product can be determined by the QR decomposition of H(k) (or $H^{w}\left(k\right)$ when a local estimation is needed). When different products are produced, the variation in H(k) significantly increases the available information, so the optimization of the production sequence can highly influence the identifiability of the model and confidence in the parameters ($P^{*}\left(N\right)$, π(k)).

**Figure 8:**
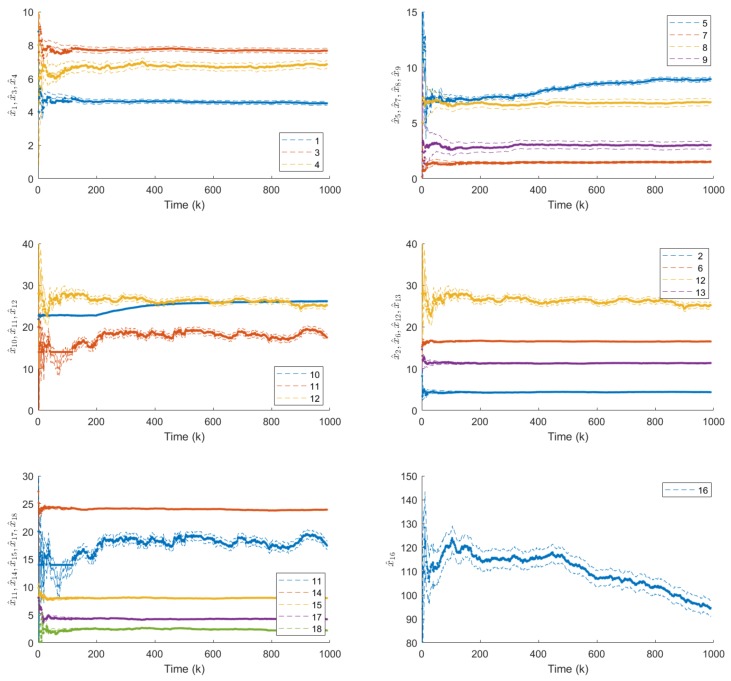
Estimated primary activity times with their p=0.01 confidence intervals (represented by dashed lines). The figure illustrates that the algorithm is able to track the changes in the $x_{10}\left(k\right)$, $x_{5}\left(k\right)$ and $x_{16}\left(k\right)$ activity times after the 200th, 300th and 500th product, respectively. The bold lines represent the constrained parameter estimates.

The production of 1000 products was studied. The production sequence contained all 64 types of products with an average batch size of 10 products/batch. The rank of the covariance matrix $F^{*}\left(N\right)$ was identical to the size of $\hat{x}\left(k\right)$, so all activities could be monitored (see Figure 8).

When the raw material, design or the processing of a component in a cost-cutting or quality-improvement project is changed by the supplier, this change may influence the activity times of the operators. Such operator-independent loss in performance can occur when a shorter length of wire increases the time required to lay and arrange the cables. In this case study, such effects are monitored. In the studied case, the new wires between the $c_{87}$ and $c_{8}$ components are a bit shorter than specified. The component $c_{87}$ (seal on the terminal) has an impact on the $t_{10}$ type of activity in the module $m_{4}$ which increases the related primary activity time ($x_{10}\left(k\right)$) by 15% at the 200th product, while the component $c_{8}$ (the shorter wire) has an impact on the activity type $t_{5}$ in the module $m_{2}$, which increases the related $x_{5}\left(k\right)$ state variable by 20% after the 300th product. In this illustrative scenario the quality inspection time decreases after the 500th product.

As Figure 8 illustrates, the proposed system is able to track the slowed and fastened activities. The benefit of the proposed constrained algorithm is clearly visible, the estimated variables converge faster and are always reliable.

The means of detecting individual losses in operator performance losses and sensor faults (due to delayed registration and IIoT communication) were also studied.

In terms of fault detection, the prediction error used in Equation (5) can be used as generates an interpretable and easily traceable univariate time series that reflects the global performance of the model.

The global performance of the model is reflected by

(19) $$e_{q}\left(k\right)={\left[z(k)-H(k)x\right]}^{T}Q\left[z(k)-H(k)x\right]\;,$$

while the local, workstation related fault detection should be based on the local observations:

(20) $$e_{q}^{w}\left(k\right)={\left[z^{w}\left(k\right)-H^{w}\left(k\right)x\right]}^{T}Q^{w}\left[z^{w}\left(k\right)-H^{w}\left(k\right)x^{w}\right]\;,$$

where $Q^{w}$ represents the *w*th block matrix of Q.

**Figure 9:**
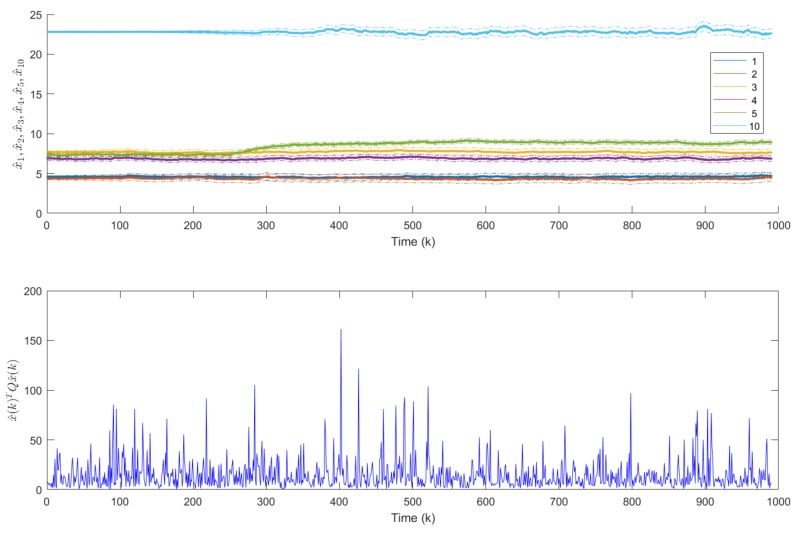
Fault-detection performance at the 2nd workstation. The upper figure illustrates that the algorithm is able to detect operator-dependent problems (after the 250th product). After this change the related to these activities.

Based on the analysis with regard to the rank of the $H^{w}\left(k\right)$ matrices, the observable sets of activities were determined. As is illustrated in Figure 9, at the w=2 workstation the time consumption of six primary activities are observable. The proposed algorithm was able not only to detect operator-dependent problems (of the 250th product) related to these activities, but by monitoring the $e_{q}\left(k\right)$ it was possible to determine when sensor faults occurred (see the bottom of the figure). The parameters of the gross error detection algorithm can be fine-tuned by Monte Carlo simulation and detailed analysis of the distribution of the modeling error [31,32] (the demonstration of the applicability of these techniques in this problem is out of the scope side this paper).

**Figure 10:**
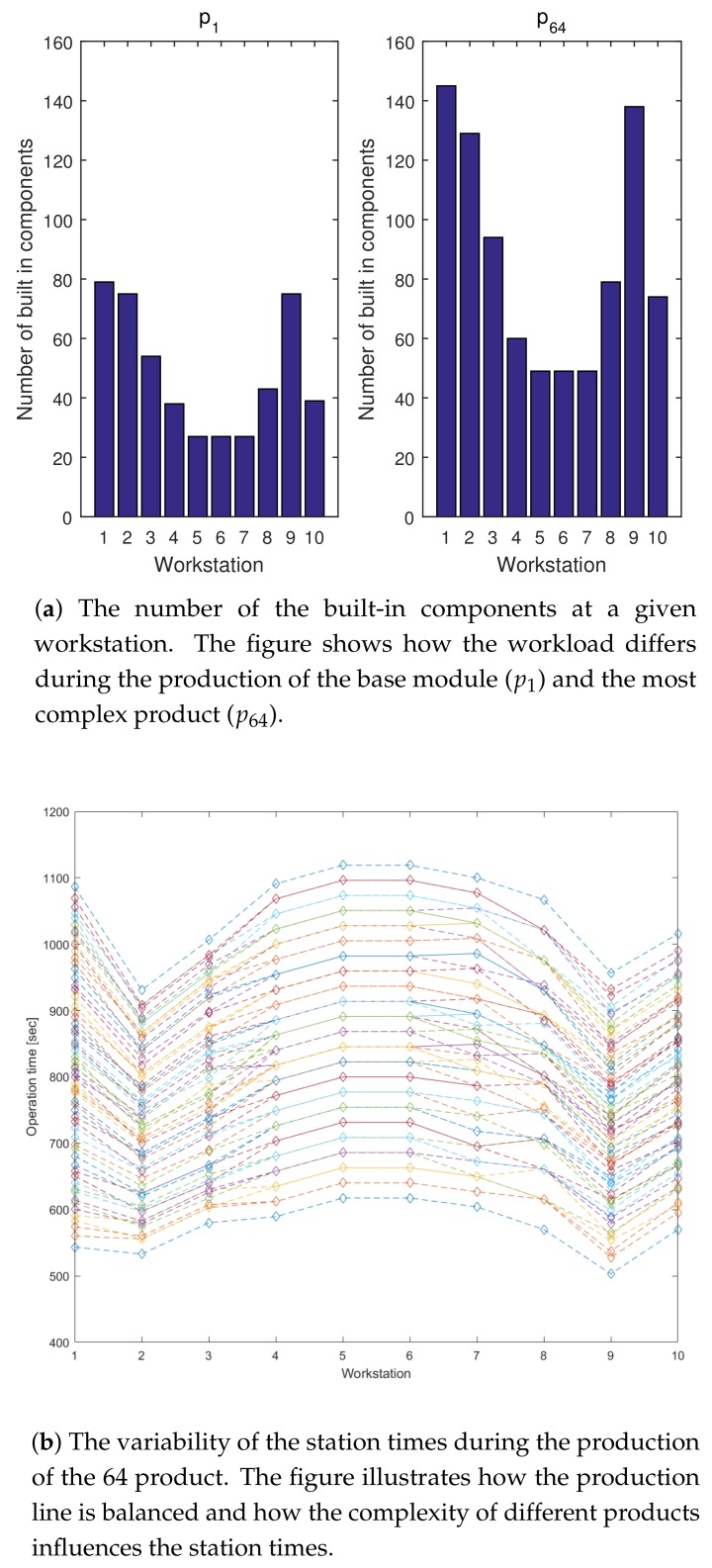
The workloads (number of activities, built-in components and total activity times) can be easily calculated based on the proposed model. The OEE of the production line can be monitored on-line based on the recursively estimated activity times.

As is illustrated in Figure 10, the calculations above can be used to estimate the expectable operation times for all workstations, check how well the process is balanced and how the complexity of the product influences the workloads of the workstations. With the help of this model the effect of the changes in the activity time can be immediately calculated on the tack-time and the effectiveness of the operators. The presented example demonstrated that in the event of good estimates with regard to the duration of the primary activities and with the help of the IIoT-based fusion of product-relevant information, real-time data for Overall Equipment Effectiveness (OEE) calculations can be provided.

### 3.2. Targeting Model-Based Workload Analysis

The monitoring of the activity of operators is based on the comparison of the measured activity and station times with the estimates of targeting models whose parameters are identified by the proposed estimation algorithms. Targeting models are widely used in process performance management. Precedent-based targeting models are used when the expected performance can be deduced from previous operations (e.g., a day or month before). One weakness of this procedure is that it assumes that conditions were comparable over the two time intervals. A more problematic issue is what happens when a significant change occurs in the process and product. For such applications precedent-based targeting models can be over simplistic. Activity-based targeting is particularly appropriate when the clear drivers of performance are known [33].

**Figure 11:**
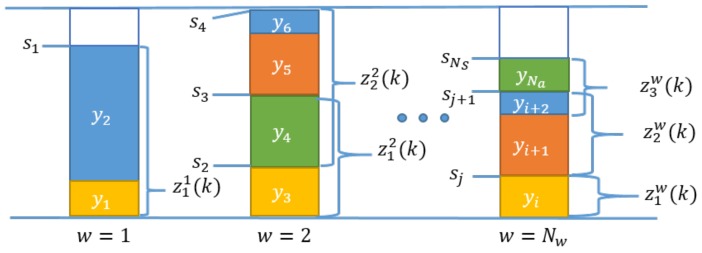
Demonstration of the distribution of the activity times $y_{i}$, the station times, and the timestamps $s_{j}$ of the fixture sensors; the measured time interval $z_{l}^{w}$; and the *w*-th workstation. The height of the bars coloured illustrates the activity time of the specific activity, while the coloured tag of the individual activities provides information on the type of the specific activity. This representation shows how the related activity times are summed up and form the station time and highlights that the longest station time is the cycle time of the production system, which is represented by the vertical blue line at the top of the figure.

Automatic monitoring and targeting schemes attempt to compare performances very short time intervals (e.g., minutes) which is ideal for fault detection. The most important key performance indicators (KPIs) of the production system are the station times which reflect how well the production line is balanced. The concept of calculating the station time is depicted in Figure 11. The balancing of a modular production system is a challenging industrial problem due to the great diversity of products [34]. As the station times are the functions of the manufactured products, which product is assembled on a given workstation must be followed. The calculation of the station time is similar to the calculation of the estimated sum of activity times between two fixture sensors (Equation (2)), namely the difference between the appropriate timestamps recorded by the fixture sensors:

(21) $$y^{w}\left(k\right)=\left[T^{T}q_{w}\;,\left(T^{T}C>0\right)C^{T}q_{w}\right]x^{w}\left(k\right)$$

where $q_{w}=diag\left(Mp_{p}^{\prime }\left(k\right)\right)W$.

## 4. Conclusions

Human-in-the-loop cyber-physical production systems are transforming the industrial workforce. Due to the enormous variability and complexity of products, the tracing of hundreds of activity times on production lines is a critical problem. To handle this problem a software-sensor-based activity-time and performance measurement system was proposed. To ensure a real-time connection between operator performance and varying degrees of product complexity fixture sensors were utilized and designed and an indoor positioning system used to merge this multi-sensor data with product-relevant information.

The presented sensor fusion algorithm combines all sensory and production data such that the estimates of the activity times have less uncertainty than would be possible when these sources were used individually. The estimation of the activity times is based on a linear-in-parameters model. The linear structure of the developed production-monitoring model is adequate as the time consumption of the activities linearly depend on how many primary activities should be performed and what is the number of the built-in components.

The number of parameters of activity time estimation models is comparable to the number the number of measurements, the identifiability of the parameters of the model has to be carefully analyzed. For this purpose, we studied the Fisher information/covariance matrix of the estimation problem. The identifiability of the model and the information content of the available data can be evaluated based on the rank, the eigenvalues and the determinant of the covariance matrix. When the rank is smaller than the number of measurements (which occurs when the individual performance of operators is estimated at a specific workstation), only a subset of the parameters is identifiable. As the placement of the sensors significantly influences the identifiability of the parameters, tools of D-optimal experimental design can be used to optimize the proposed system.

The determination of the optimal number of sensors and features has crucial importance as redundant sensors can generate correlated features which decrease the efficiency of the algorithm. The analysis of the eigenvalues of the covariance matrix can highlight these negative effects. As this analysis is identical to Principal Component Analysis (PCA) of the multisensor data, the proposed methodology can utilize the reduced and transformed uncorrelated features, which results in a Principal Regression-based process monitoring algorithm. The second approach of avoiding correlated features is the application of feature selection algorithms that should be based on the previously discussed experimental design optimization task.

As the estimation problem can be ill-conditioned and poor raw sensor data can result in unrealistic parameter estimates, constraints were introduced into the parameter-estimation algorithm to increase the robustness of the software sensor.

The proposed model-based performance monitoring system tracks the recursively estimated parameters of the activity-time estimation models, while the sensor-relevant fault detection functionalities are based on the modeling errors which can be evaluated by classical residual-based fault detection algorithms.

The applicability of the proposed methodology is demonstrated on a well-documented benchmark problem of a wire harness manufacturing process. The presented example demonstrated the benefits of multiple sensors as they provide redundancy which enables the robust recursive estimation of the unmeasured primary activity times. The fully reproducible and realistic simulation study also confirmed the efficiency of the proposed constrained estimation algorithm regarding fast convergence and giving reliable estimates.

The results illustrate that indoor positioning system-based integration of product-relevant information and sensor signals and can be efficiently utilized to design on-line performance management systems.

The developed benchmark problem can be used to study fault detection and sensor placement algorithms which is the objective of our further research.

## Figures and Tables

**Table 1:** The logical matrices defined for performance monitoring.

Notation	Nodes	Description	Size
A	product (p) - activity (a)	activity required to produce a product	$N_{p}\times N_{a}$
W	activity (a) - workstation/machine (w)	workstation assigned for an activity	$N_{a}\times N_{w}$
B	product (p) - component/part (c)	component/part required to produce a product	$N_{p}\times N_{c}$
P	product( p) - module ( m)	module/part family required to produce a product	$N_{p}\times N_{m}$
C	activity (a) - component (c)	component/part built in or processed in an activity	$N_{a}\times N_{c}$
M	activity (a) - module (m)	activity required to produce a module	$N_{a}\times N_{m}$
T	activity (a) - activity type (t)	category of the activity	$N_{a}\times N_{t}$
$S^{w}$	activity (a) - measured time interval ($z^{w}\left(k\right)$)	activity involved over a measured time interval	$N_{a}\times l_{w}$

**Table 2:** Types of activities and the related activity times according to [15]. The activity times are calculated using a direct proportionality approach, e.g., when an operator is laying four wires over one foot, proportionally to the parameter $t_{4}$, the activity time will be 1 × 6.9 s + 4 × 4.2 s = 23.7 s.

ID	Activity	Unit	Time [s]
$t_{1}$	Point-to-point wiring on chassis	Number of wires	4.6
$t_{2}$	Laying in U-channel		4.4
$t_{3}$	Laying flat cable		7.7
$t_{4}$	Laying wire(s) onto harness jig	Per wire	6.9
			4.2
$t_{5}$	Laying cable connector (one end) onto harness jig	Per wire	7.4
			2.3
$t_{6}$	Spot-tying onto cable and cutting		16.6
$t_{7}$	Lacing activity		1.5
$t_{8}$	Taping activity		6.8
$t_{9}$	Inserting into tube or sleeve		3.0
$t_{10}$	Attachment of wire terminal		22.8
$t_{11}$	Screw fastening of terminal		17.1
$t_{12}$	Screw-and-nut fastening of terminal		24.7
$t_{13}$	Circular connector		11.3
$t_{14}$	Rectangular connector		24.0
$t_{15}$	Clip installation		8.0
$t_{16}$	Visual testing		120.0

**Table 3:** The placement of the sensors is defined based on the activity IDs. As can be seen in the table, not all the $f_{i}$i=1,…,16 fixtures are active at every $w_{j}$j=1,…,10 workstation.

Sensor ID	$w_{1}$	$w_{2}$	$w_{3}$	$w_{4}$	$w_{5}$	$w_{6}$	$w_{7}$	$w_{8}$	$w_{9}$	$w_{10}$
$f_{1}$	1	79	159							
$f_{2}$	12	90	170							
$f_{3}$	21	99	175							
$f_{4}$	31	109	181							
$f_{5}$	44	121	185	226						
$f_{6}$								422	486	595
$f_{7}$								438	514	603
$f_{8}$								448	535	
$f_{9}$								451	540	615
$f_{10}$		132	192		275	324	373	453		
$f_{11}$					323	372		482		
$f_{12}$							419			
$f_{13}$										617
$f_{14}$										630
$f_{15}$									547	
$f_{16}$										654

## Supplementary Materials

The related dataset is freely and fully available on the website of the authors: https://www.abonyilab.com/soft-sensors.

## Notations and Abbreviations

**CPPS**	Cyber-physical production system
**IIoT**	Industrial Internet of Things
**RFID**	Radio Frequency IDentification
**BoM**	Bill of Materials
**IPS**	Indoor positioning system
**UWB**	Ultra-Wide Band
**BLE**	Bluetooth Low Energy
**MSDF**	Multi-sensor data fusion
**AoA**	Angle of Arrival
**SS**	Signal Strength
**RSS**	Received Signal Strength
**ToA**	Time of Arrival
$p_{1},\ldots ,p_{N_{p}}$	products
$m_{1},\ldots ,m_{N_{m}}$	modules
$a_{1},\ldots ,a_{N_{a}}$	activities
$c_{1},\ldots ,c_{N_{c}}$	components
$w_{1},\ldots ,w_{N_{w}}$	workstations
$t_{1},\ldots ,t_{N_{t}}$	activity types
A	($N_{p}\times N_{a}$) activities required to produce a product
W	($N_{a}\times N_{w}$) workstation assigned for an activity
B	($N_{p}\times N_{c}$) component/part required to produce a product
P	($N_{p}\times N_{p}$) module/part family required to produce a product
C	($N_{a}\times N_{c}$) component/part built in or processed in an activity
M	($N_{a}\times N_{m}$) activity required to produce a module
T	($N_{a}\times N_{t}$) category of the activity
$S^{w}$	($N_{a}\times l_{w}$) activity involved over a measured time interval
*k*	index of the production cycle (discrete time)
${\hat{y}}_{i}^{w}\left(k\right)$	estimation of the individual activity times for work station *w* in the *k*th production cycle
$x^{w}\left(k\right)$	’efficiency’ of the operator, the vector of the estimated local activity times
x(k)	workstation-independent version of $x^{w}\left(k\right)$
s(k)	sequence of the timestamps recorded by the active fixture sensors
$z^{w}\left(k\right)$	vector of the sum of the activity times that are situated between the two sensors
α	index of the first sensor of a fixture-sensor pair
β	index of the second sensor of a fixture-sensor pair
$q_{a}$	the set of activities required to produce a specific product
$e^{w}$	serially uncorrelated white-noise vector of observational errors
$R^{w}\left(k\right)$	covariance matrix of observational errors
H(k)	time-variable regressors representing the number of activities and built in components
e(k)	the set of the serially uncorrelated white-noise vector of observational errors of the workstations
$\hat{x}\left(N\right)$	estimation error
Q	positive-definite weighting matrix defined as $Q={\left(R\right)}^{-1}$
$P^{*}$	inverse of the parameter covariance matrix
$A^{*}\left(k\right)$	State-transition matrix in the Kalman filter represented estimation problem (in our case an identity matrix)
K(k)	Gain of the Kalman filter/recursive estimator
$L^{w}$,$c^{w}$	Representation of the linear inequality constraints
$A_{e}^{w}$,$b_{e}^{w}$	Representation of the linear equality constraints
$\mu_{j}$	vector of Lagrange multiplier associated with equality
$\lambda_{j}$	vector of Lagrange multiplier associated with inequality constraints
